# Modification of immune cell-derived exosomes for enhanced cancer immunotherapy: current advances and therapeutic applications

**DOI:** 10.1038/s12276-023-01132-8

**Published:** 2024-01-04

**Authors:** Inseong Jung, Sanghee Shin, Moon-Chang Baek, Kyungmoo Yea

**Affiliations:** 1 Department of New Biology, DGIST, Daegu, 42988 Republic of Korea; 2 Department of Molecular Medicine, CMRI, Exosome Convergence Research Center (ECRC), School of Medicine, Kyungpook National University, Daegu, 41944 Republic of Korea; 3 New Biology Research Center, DGIST, Daegu, 43024 Republic of Korea

**Keywords:** Cancer immunotherapy, Nanoparticles

## Abstract

Cancer immunotherapy has revolutionized the approach to cancer treatment of malignant tumors by harnessing the body’s immune system to selectively target cancer cells. Despite remarkable advances, there are still challenges in achieving successful clinical responses. Recent evidence suggests that immune cell-derived exosomes modulate the immune system to generate effective antitumor immune responses, making them a cutting-edge therapeutic strategy. However, natural exosomes are limited in clinical application due to their low drug delivery efficiency and insufficient antitumor capacity. Technological advancements have allowed exosome modifications to magnify their intrinsic functions, load different therapeutic cargoes, and preferentially target tumor sites. These engineered exosomes exert potent antitumor effects and have great potential for cancer immunotherapy. In this review, we describe ingenious modification strategies to attain the desired performance. Moreover, we systematically summarize the tumor-controlling properties of engineered immune cell-derived exosomes in innate and adaptive immunity. Collectively, this review provides a comprehensive and intuitive guide for harnessing the potential of modified immune cell-derived exosome-based approaches, offering valuable strategies to enhance and optimize cancer immunotherapy.

## Modified Exosomes: Amplifying Immune Response for Tumor Control

The study investigates the use of tiny particles released from the immune system, known as immune cell-derived exosomes (IEXs), for cancer treatment. This research aspires to bridge the existing knowledge gap about engineering these IEXs to boost their inherent anti-cancer properties. The team of researchers, headed by PhD student, Inseong Jung and Sanghee Shin, and Professors Kyungmoo Yea and Moon-Chang Baek, investigated using gene alteration and physical and chemical changes to enhance the therapeutic potential of IEXs. The study included testing these altered IEXs on different immune and cancer cells, with the results indicating hopeful outcomes for cancer treatment. The researchers conclude that while these IEXs have substantial potential, further refinement and optimization are required to overcome limitations and enhance their clinical use. They also underline the potential of this method for future cancer-fighting strategies.

This summary was initially drafted using artificial intelligence, then revised and fact-checked by the author.

## Introduction

Cancer immunotherapy has gained widespread attention for its ability to activate the immune system to target tumors, unlike conventional treatments that affect both cancerous and healthy cells^1^. The success of immunotherapy highlights the crucial role of the immune system in the elimination of malignant cells^2^. Tumor growth is precisely controlled by two major immune cell groups: innate and adaptive immune cells^3^. Innate immune cells destroy tumor cells to induce the release of tumor-specific antigens. They are captured by antigen-presenting cells (APCs), including dendritic cells (DCs), and presented to T cells to prime the adaptive immune response. Subsequently, antigen-specific T cells evoke a specific immune response against cancer cells^4,5^. Cancer immunotherapies that harness the power of this immune system can specifically kill cancer cells with minimal influence on normal cells and trigger long-term memory that prevents tumor recurrence^5^. Thus, the development of methods to direct or harness the immune response can enhance the success of cancer immunotherapy.

In recent decades, the proposal of various new immunotherapies has emerged, with treatment strategies being continuously refined to improve antitumor efficacy^6,7^. Monoclonal antibody-based immune checkpoint blockades (ICBs) have shown substantial antitumor activity in several cancers, such as melanoma, lung cancer, and kidney cancer^7,8^. However, the clinical effect of this approach is only beneficial in approximately 20–30% of patients, with the majority showing resistance^7^. Chimeric antigen receptor (CAR)-T cell therapy, a treatment that genetically engineers a patient’s T cells to express CARs targeting specific cancer cells, exhibits significant efficacy in the treatment of hematologic malignancies, including acute lymphocytic leukemia and non-Hodgkin lymphoma^6,7,9^. However, the use of this therapy in solid tumors has been impeded by tumor microenvironmental barriers and immunosuppressive environments, and CAR-T cells have elicited severe toxicities, such as cytokine release syndrome (CRS)^9^. Therefore, the field of immunotherapy is currently aspiring to discover novel, low-toxicity, and biostable immunomodulators.

Emerging evidence indicates that exosomes, ranging in size from 30 to 150 nm, can modulate the antitumor immune response, presenting them as a promising avenue for cancer immunotherapy^10^. As endogenous transporters, exosomes deliver lipids, proteins, and nucleic acids to nearby or distant cells^11^. Compared to conventional nanoparticles, such as viruses and synthetic nanocarriers, endogenously derived exosomes demonstrate superior biocompatibility, evade phagocytosis, and significantly reduce immunogenicity^12^. Specifically, exosomes can readily infiltrate the extracellular matrix of tumor tissue, unaffected by the tumor microenvironment (TME), thereby overcoming the challenges of cell therapy^13^. Owing to these properties of exosomes, immune cell-derived exosomes (IEXs) have emerged as key immune response modulators that can regulate the TME and even directly shape antitumor activity^4,13,14^. Given these unique and valuable biological and pharmacological properties, IEXs have attracted considerable scientific interest as a novel strategy for cancer immunotherapy.

Importantly, IEXs can be designed to acquire therapeutic potential and improve the efficacy of existing agents^15^. In recent years, various exosome modification strategies, including genetic engineering, chemical modification, and cargo transport, have been developed to boost their therapeutic potential^16,17^. Additionally, preconditioning of parental cells with cytokines that augment immune cell function can enhance the secretion or antitumor efficacy of their exosomes^18^. We propose that exosomes that trigger a higher level of immune response against cancer through various manipulations can pave the way for novel directions in cancer immunotherapy.

In this review, the different strategies employed to design exosomes are investigated, and the recent therapeutic applications of modified innate and adaptive IEXs are discussed. The potential of these modified exosomes to serve as an innovative tool in the fight against cancer inspires further research and development in cancer immunotherapy.

## Modification strategies of IEXs for cancer immunotherapy

**Fig. 1:**
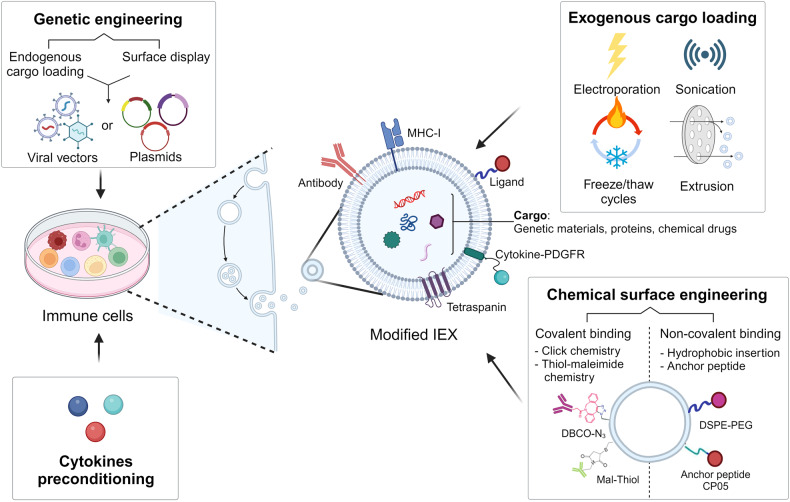
Modification strategies of IEXs for cancer immunotherapy. IEXs can undergo modifications to either encapsulate therapeutic molecules or display them on the IEX surface. Pretreatment of immune cells with cytokines enhances IEX efficacy. Genetic engineering, using viral vectors or plasmids, packages therapeutics inside IEXs or expresses molecules such as antibodies on the IEX surface. Physical methods, such as electroporation, sonication, freeze‒thaw cycles, and extrusion, increase the permeability of the exosome membrane to facilitate the loading of drugs. Chemical modifications, both covalent and noncovalent, allow for the display of ligands or receptors on the IEX surface. Created with BioRender.com.

With the increase in the understanding of the biological properties of IEXs, there is a growing interest in research aimed at manipulating these exosomes to optimize their therapeutic potential^16–20^. A wide range of techniques have been employed for such modifications, from parental cell preconditioning to cargo packaging and surface engineering (Fig. 1). These modifications have revealed promise for cancer treatment by improving the target specificity, immunogenicity, biodistribution, and pharmacokinetics of exosomes.

### Parental cell preconditioning

Cytokines are important factors in the stimulation of the immune system to regulate immune cell activity^21^. Growing evidence suggests that cytokine pretreatment of parental cells can promote IEX activity to enhance therapeutic efficacy^22–26^. DC-derived exosomes (DEXs) preconditioned with interferon-γ (IFN-γ), which stimulates immature DC differentiation into mature DCs, augmented natural killer (NK) cell-mediated antitumor efficacy against non-small cell lung cancer (NSCLC) patients^22^. Similarly, IFN-γ-treated macrophages exhibited an M1 phenotype, and exosomes derived from these cells migrated to the lymph nodes, creating a proinflammatory microenvironment^23^. Interleukin (IL)-15 and IL-21-stimulated NK cell-derived exosomes possessed profound cytotoxic activity^24^. Furthermore, it was demonstrated that CD4^+^ T cell-derived exosomes pretreated with IL-2, a key regulator of T cells, increase the cytotoxicity of CD8^+^ T cells^25^. Li et al. elucidated that exosomes from CD8^+^ T cells cultured with IL-12 can promote the proliferation of bystander resting CD8^+^ T cells and the production of IFN-γ and granzyme B^26^. Thus, IEXs generated from cytokine-pretreated parental cells appear to be a step-up from natural exosomes. With the absence of specialized equipment requirements and cost efficiency, this approach holds considerable promise in clinical applications.

### Cargo packaging into exosomes

Exosomes have emerged as an attractive drug delivery system (DDS) due to their low toxicity, high biocompatibility, and stability^5,17,19,20^. Cargo can be packaged by endogenous and exogenous loading methods into exosomes^27^. Endogenous cargo loading is typically performed through parental cell modification using viral vectors and plasmids that are commonly employed for nucleotide or protein loading^19,27^. Kaban et al. utilized lentiviruses produced from HEK293T cells to load BCL-2 siRNA into NK cell-derived exosomes. This process allows specific siRNAs to be overexpressed in NK cell-derived exosomes, inducing breast cancer cell apoptosis^28^. However, despite the high loading efficiency, toxicity and stability issues persist, and there are concerns regarding the alterations in the biological activity of the exosomes due to modifications of gene expression in the producing cells^20^. Exogenous cargo loading employs techniques such as electroporation, sonication, freeze‒thaw cycles, extrusion, and saponin treatment to load small-molecule drugs^5,19,20^. For instance, electroporation creates small pores in the exosome membrane through an electric field, facilitating drug diffusion into the exosome^19,20^. The integrity of the exosome membrane is restored after drug loading^17^. Han et al. successfully loaded the antitumor drug paclitaxel (PTX) into exosomes isolated from NK cells using electroporation to increase apoptosis of MCF-7 cells^29^. However, the electroporation process can potentially compromise the stability of exosomes, resulting in a diminished loading capacity^19^. An alternative method, sonication, facilitates drug permeation by decreasing the microviscosity of the exosomal membrane^19^. Using sonication, PTX was loaded into exosomes derived from the M1 phenotype polarized via IFN-γ. These M1 exosomes transported PTX into the tumor tissue and accelerated its antitumor effects^30^. However, it is important to note that sonication can impose a certain degree of membrane damage^5^. Although the strategies to engineer exosomes still show limitations, drug delivery through the manipulation of these IEXs has successfully inhibited cancer cells while also reducing side effects and enhancing treatment outcomes.

### Genetic engineering of the exosome surface

Exosomes exhibit stable circulation within the bloodstream, but they present challenges in targeting specific cells^31^. Several strategies have been proposed to improve tumor-specific targeting in exosome-based cancer immunotherapy^17,20,31^. Strategies for exosome surface engineering have been divided into genetic engineering and chemical modification, and these approaches allow tumor tissue-specific therapy^17,20,31^. Genetic engineering approaches are useful for displaying targeted ligands on the exosome membrane. Parental cells can express the desired membrane-bound protein via lentiviral packaging techniques or transfection with a plasmid encoding the gene of interest^32–34^. Overexpressed proteins in donor cells subsequently confer modifications to the membrane surface of exosomes secreted by these cells. An illustrative example is DCs, which were transfected with the plasmid vector pcDNA3.1(+) encoding the anti-programmed cell death protein-1 (PD-1) single-chain variable fragment gene, enabling the engineering of membrane-localized anti-PD-1 antibodies. Exosomes isolated from these modified cells then reinvigorate exhausted CD8^+^ T cells, thereby improving cytotoxic T lymphocyte (CTL) responses^32^.

Recently, membrane-tethering technology for proteins (MTFP) has been introduced as a promising strategy to display bioactive proteins, such as cytokines and antibodies, on the cell surface^21^. MTFP expresses cytokines and antibodies via a glycine-serine linker with the transmembrane domain of the platelet-derived growth factor receptor (PDGFR) on the exosome membrane. Additionally, the linker provides flexibility to allow an increase in accessibility to neighboring receptor molecules. Remarkably, cytokines expressed in cells with MTFP exert an autocrine effect, triggering continuous downstream signaling of receptors in a membrane-anchored form. Intriguingly, IL-2 surface tethering on T cells using MTFP facilitated the self-activation of T cells and caused a dramatic enrichment of antitumor miRNAs within exosomes derived from these T cells. IL-2 tethering by MTFP allowed both internal reprogramming of exosomes and external drug delivery, thus enhancing the antitumor efficacy of exosomes^33^. Moreover, CD8^+^ T cell-derived exosomes tethered with both anti-epidermal growth factor receptor (EGFR) antibodies and IL-2 via MTFP specifically induced EGFR-positive cancer cell death through immune activation^34^. These genetic modification strategies for IEXs represent a viable avenue for the creation of novel targeted therapeutics.

### Chemical modification of the exosome surface

IEXs can undergo chemical alterations to display both natural and synthetic ligands on their surface. Chemical conjugation encompasses both covalent and noncovalent modifications^17,31^. The direct attachment of antigens or other molecules to exosomes using click chemistry has been used as an effective covalent surface engineering method. Click chemistry is a copper-catalyzed azide-alkyne cycloaddition reaction that facilitates a bioorthogonal bond between an azide and an alkyne group^35^. However, owing to the cytotoxic effects of copper, a strain-promoted azide-alkyne cycloaddition through a strained cyclooctyne has been developed as a copper-free reaction^35^. In this approach, azide-modified M1 macrophage-derived exosomes were simultaneously conjugated with dibenzocyclooctyne (DBCO)-modified anti-CD47 and anti-SIRPα antibodies via benzoic-imine linkages. These pH-sensitive benzoic-imine bonds were cleaved in the acidic TME. This leads to the release of anti-SIRPα and anti-CD47 antibodies, thereby increasing tumor cell phagocytosis^36^. Unlike covalent modifications, noncovalent coupling, such as electrostatic and hydrophobic interactions, is a strategy for the stable modification of biological membranes^17,31^. The lipid bilayer membrane of exosomes facilitates the spontaneous incorporation of amphiphilic substances via hydrophobic interactions. Kim et al. successfully conjugated aminoethyl anisamide (AA), a sigma ligand, into exosome membranes based on polyethylene glycol (PEG)-grafted 1,2-distearoyl-sn-glycero-3-phosphoethanolamine (DSPE-PEG). These engineered exosomes exhibited high targeting efficacy for sigma receptors, which are overexpressed in lung cancer, and accumulated efficiently in cancer cells upon systemic administration, leading to prolonged survival in a lung metastasis mouse model^37^. Furthermore, DSPE-PEG is an FDA-approved material for medical use that can be widely applied to exosome membrane modification to enhance its clinical potential^17^.

Overall, to address their limitations alongside technological advances, current strategies for exosome modifications have continuously evolved. These challenges can be sequentially addressed through multifaceted manipulations, and the integration of these diverse engineering strategies can potentiate robust antitumor efficacy, offering a novel and innovative approach to cancer immunotherapy.

## Modification of innate IEXs

**Fig. 2:**
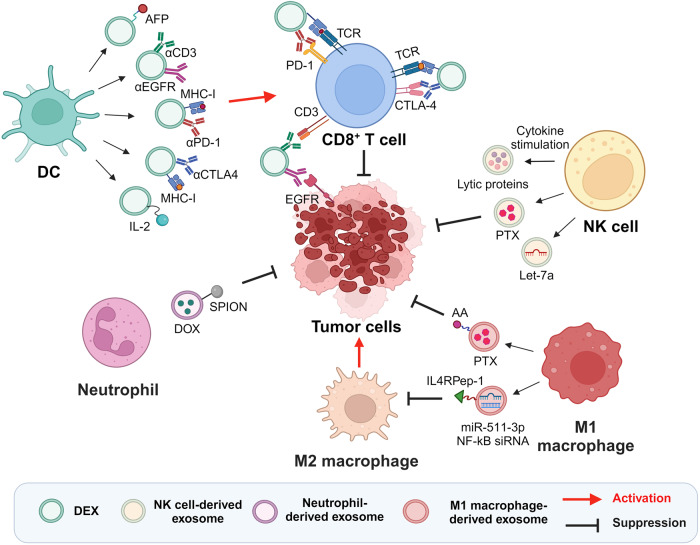
Schematic diagram illustrating the function of modified innate IEXs in the TME. Modified innate IEXs affect various immune cells and cancer cells within the TME. DEXs engineered with tumor-derived antigens, T cell-targeting antibodies, and cytokines activate CD8^+^ T cells. NK cell-derived exosomes exert cytotoxic effects through enhanced lytic proteins due to cytokine stimulation or mediate cell death via delivery of chemical drugs or antitumor miRNAs. M1 macrophage-derived exosomes amplify antitumor response by directly killing cancer cells via drug delivery or promoting M2-to-M1 macrophage polarization. Neutrophil-derived exosomes equipped with SPIONs and loaded with DOX target tumor and exhibit tumor suppressive activity. Created with BioRender.com.

Exosomes from innate immune cells, including DCs, NK cells, macrophages, and neutrophils, are vital in immune surveillance and response^18^. These exosomes carry the immunological traits of their parent cells and can interact with other immune cells. To amplify their inherent antitumor properties, they can also be engineered, making them promising for cancer immunotherapy^16^. In the following section, we discuss the unique characteristics of each type of engineered innate IEX, as uncovered by recent research (Fig. 2 and Table 1).

**Table 1:** Application of modified innate IEXs for enhancing cancer treatment.

Source	Type of modification	Molecule involved in modification	Target cancer	Function	Reference
DCs	Incubation	OVA	B16/OVA	- Activation of B cells, CD4^+^ T cells, and OVA-specific CD8^+^ T cells	^48^
	Incubation	B16 antigen and poly (I:C)	B16F10	- Activation of melanoma-specific CD8^+^ T cells - Recruitment of NK and NK-T cells into tumors	^49^
	Lentiviral infection	AFP gene	HCC	- Increase in IFN-${\rm{\gamma }}$-expressing CD4^+^ and CD8^+^ T cells - Reduction in IL-10 and TGF-β in TME	^51^
	Conjugation using CP05 anchor	HCC-targeting peptide (P47-P), an AFP epitope (AFP212-A2), and a functional domain of high mobility group nucleosome-binding protein 1 (N1ND-N)	Hepa1-6	- Activation of DCs - Recruitment of DCs into tumors	^52^
	Incubation and conjugation using DSPE-PEG-NHS	OVA (incubation) and anti-CD3 and EGFR antibodies (conjugation)	B16/OVA	- Activation of endogenous T cells in lymph node and spleen - Connection between T cells and cancer cells	^53^
	Transfection and adenoviral infection	Anti-PD-1 antibody (transfection) and MHC-I-antigen complex (infection)	Hepa1-6-OVA, B16F10, LLC, and MC-38	- Presentation of cancer antigens - Activation of cancer-specific CTL response	^32^
	Incubation and conjugation using Sulfo-SMCC	Gp100 peptides (incubation) and anti-CLLA-4 antibody (conjugation)	B16F10	- Increase in antigen-specific CD8^+^ T cell activity - Recovery of exhausted CD8^+^ T cells	^54^
	Lentiviral infection	IL-2-MFG-E8	EO771 and 4T1	- Priming and activation of T cells in the spleen and lymph node	^55^
NK cells	Incubation	IL-15 and IL-21	K562, Jurkat, A549, and HeLa	- Increase in cytolytic activity against cancer cells	^24^
	Incubation	IL-15	U87/MG, MDA-MB-231, and CAL-62	- Increase in tumor-targeting ability - Induction of apoptosis in cancer cells	^61^
	Incubation	IL-12, IL-15, and IL-18	HCT116, HCT-15, DU145, PC3, SK-BR-3, T-4D7, OVCAR-3, WM9, and U87	- Induction of apoptosis in cancer cells	^62^
NK cells	Electroporation	PTX	MCF-7	- Reduction in migration of cancer cells - Induction of apoptosis in cancer cells	^29^
	DBCO linked with Ph-sensitive benzoic-imine	CTLs	B16/OVA	- Facilitation of tumor-targeting accumulation of CTLs - Induction of apoptosis in cancer cells - Upregulation of MHC-I expression on cancer cell surface	^63^
	Incubation	Let-7a-loaded-dendrimers	MDA-MB-231 and CHLA-255	- Tumor-specific accumulation of the exosomes - Induction of apoptosis in cancer cells - Delivery of miRNAs into tumor	^64^
Macrophages	Sonication	PTX	4T1	- Induction of apoptosis in cancer cells	^30^
	Sonication and conjugation using DSPE-PEG-NHS	PTX (sonication) and AA-PEG (conjugation)	3LL-M27	- Increase in circulation time in blood - Delivery of PTX into target cancer cells - Eradication of pulmonary metastases	^37^
	Transfection and conjugation using DOPE-PEG-NHS	NF-$\kappa$B p50 siRNA and miR-511-3p (transfection), and IL4RPep-1 (conjugation)	4T1 and LLC	- Increase in circulation time in blood - Enhancement of targeting M2 macrophages - Downregulation of NF-$\kappa$B p50 and ROCK2 expression in M2 macrophages	^74^
	Incubation	Tumor cell nuclei	E.G7, 4T1, and B16	- Activation of DCs and macrophages - Augmentation of T cell proliferation and activation	^75^
Neutrophils	Sonication	DOX	C6 glioma	- BBB penetration - Enhancement of tumor-targeting ability - Inhibition of tumor proliferation	^78^
	Extrusion and Tf-TfR interaction	DOX (extrusion) and SPION (Tf-TfR interaction)	GES-1, HGC27, HepG2, and SW480	- Enhancement of tumor-targeting ability - Induction of cytotoxicity against cancer cells - Inhibition of cancer cell proliferation	^79^

*DCs* dendritic cells, *OVA* ovalbumin, *AFP* α-fetoprotein, *HCC* hepatocellular carcinoma, *IFN-γ* interferon-γ, *IL-10* interleukin-10, *TGF-β* transforming growth factor-β, *TME* tumor microenvironment, *DSPE-PEG-NHS* 1,2-distearoyl-sn-glycero-3-phosphoethanolamine-polyethylene glycol-succinimidyl ester, *EGFR* epidermal growth factor receptor, *PD-1* programmed cell death protein-1, *MHC-I* major histocompatibility complex class-I, *CTL* cytotoxic T lymphocyte*, Sulfo-SMCC* sulfosuccinimidyl-4-(*N*-maleimidomethyl)cyclohexane-1-carboxylate, *Gp100* glycoprotein 100*, CTLA-4* cytotoxic T lymphocyte associated protein-4, *NK cells* natural killer cells, *MFG-E8* milk fat globule-epidermal growth factor-factor 8, *DOX* doxorubicin, *PTX* paclitaxel, *DBCO* dibenzocyclooctyne, *AA-PEG* anisamide-polyethylene glycol, *DOPE* 1,2-dioleoyl-sn-glycero-3-phosphoethanolamine, *ROCK2* rho-associated coiled-coil containing protein kinase 2, *BBB* blood-brain barrier, *Tf* transferrin, *TfR* transferrin receptor, *SPION* superparamagnetic iron oxide nanoparticle.

### DEXs

DCs are the most effective APCs, initiating antigen-specific immune responses and acting as a bridge between innate and adaptive immune responses. In the TME, DCs present tumor-specific antigens through major histocompatibility complex class-I (MHC-I) and -II (MHC-II) molecules to effector T cells in cooperation with accessory costimulatory molecules such as CD80 and CD86^38,39^. Since a study demonstrated the ability of DEX to express functional MHC-I and MHC-II molecules and induce tumor regression via CTL activation, DEXs have become a significant cancer immunotherapy strategy^40,41^. The presence of C-C motif chemokine receptor 7 molecules on the DEX membrane allows them to migrate to T cells in the spleen, and they can express intracellular adhesion molecule-1 (ICAM-1) to promote recruitment to T cells through binding to lymphocyte function-associated antigen-1, a surface mediator of T cells^42,43^. Additionally, DEXs directly activate NK cells in MHC-independent manners, including through IL-15Rα, tumor necrosis factor (TNF) superfamily ligand, and natural killer Group 2 member D (NKG2D) on their surface^44,45^.

The capacity of DEXs to enhance antitumor immune responses is amplified after maturation stimuli. For instance, exosomes from lipopolysaccharide-induced mature DCs showed 50–100-fold more potent antigen-specific T cell activation than exosomes from immature DCs^46^. These mature DEXs exhibited notably high expression of miR-155^47^. Moreover, DEXs obtained after incubation with ovalbumin (OVA), a representative tumor-specific antigen, demonstrated the ability to activate both CD4^+^ T cells and B cells, leading to enhanced CTL responses^48^. DEXs, stimulated using the Toll-like receptor 3 agonist poly (I:C) and loaded with the B16 antigen, proliferated effector CD8^+^ T cells and recruited NK and NK-T cells to tumors in melanoma-bearing animals^49^. DEXs can offer better stability and easier manipulation than DCs^50^, leading to various efforts to boost their therapeutic effect. Particularly, in an orthotopic hepatocellular carcinoma (HCC) mouse model, DEXs engineered to express the HCC antigen α-fetoprotein (AFP) revealed a strong antitumor response by reducing immunosuppressive cytokines, such as IL-10 and transforming growth factor (TGF)-β, and increasing IFN-γ-expressing CD8^+^ T cells^51^. These results underscore the potential of engineered DEXs as cell-free vaccines for HCC immunotherapy. Further investigations involving DEXs engineered with HCC-targeting peptides, AFP, and immune adjuvants (DEX_P&A2&N_) to promote the recruitment and activation of DCs demonstrated complete tumor eradication in orthotopic HCC mice by activating both innate and adaptive immunity^52^. Notably, in light of the limited potency of ICBs, a cornerstone of immunotherapy, several therapeutic modifications were applied to DEXs^32,53–55^. Fan et al. developed DC exosomes engineered with anti-CD3 and anti-EGFR antibodies. Precultured with OVA, modified DEXs activated T cells via MHC-antigen peptide complexes and CD86 costimulatory molecules and acted as a bridge between cancer cells and T cells by simultaneously targeting T cell surface CD3 and EGFR on cancer cells. Surprisingly, these exosomes effectively inhibited tumor recurrence and metastasis by upregulating programmed death-ligand 1 (PD-L1) expression in tumors, reducing the immune escape of tumor cells when combined with ICB^53^. Liu et al. proposed an alternative therapy for anti-PD-1 antibodies through the direct expression of anti-PD-1 antibodies on DEXs. These engineered exosome platforms integrating antigen self-presentation and immune suppression reversal (ASPIRE) can directly present neoantigens to CD8^+^ T cells and have shown the potential to overcome immune tolerance by enhancing the immune function of anti-PD-1 antibodies and CD28/B7 costimulation^32^. Additionally, Jung et al. produced DEXs carrying both anti-cytotoxic T lymphocyte associated protein (CTLA)-4 antibodies and tumor antigens to improve the response rate of anti-CTLA-4 antibodies and reduce toxicity. These exosomes prevented side effects by exclusively activating tumor-specific CTLs by blocking CLTA-4 while presenting tumor antigens^54^. To treat immunotherapy-resistant breast cancer, IL-2-tethered exosomes derived from personalized autologous DCs pulsed with patient tumor lysates sensitized tumor cells to ICBs through lymphocyte targeting and IL-2-mediated immune cell activation^55^. These multipurpose DEXs with more than one modification showed synergistic effects of activating immune responses and inhibiting immune escape. Such a strategy holds significant value as a combination immunotherapy platform capable of augmenting the efficacy of ICB therapy.

### NK cell-derived exosomes

NK cells, as an integral component of the innate immune response, contribute to adoptive immunotherapy and exhibit functional activity against metastatic or hematological cancers^56,57^. In addition to direct utilization of NK cells, recent studies have revealed the remarkable antitumor efficacy of NK cell-derived exosomes^58^. Exosomes from these cells possess typical NK cell components, including perforin, granzymes, Fas ligand (FasL), and granulysin^59^. By utilizing the well-established direct killing pathway, these exosomes effectively eliminate cancer cells, such as melanoma and breast cancer cells^59,60^.

In addition to their cytotoxic effects, there is a growing body of literature elucidating various modifications aimed at enhancing their antitumor activity in cancer immunotherapy. Exosomes derived from IL-15- and IL-21-activated NK-92 cells revealed enhanced cytolytic activity against cervical or lung cancer^24^. Zhu et al. discovered that exosomes derived from IL-15-primed NK-92MI cells exhibited notably increased cytotoxicity against various human cancer cell types, such as glioblastoma, breast cancer, and thyroid cancer cells^61^. Moreover, exosomes released by IL-12-, IL-15-, and IL-18-stimulated NK cells, including primary NK cells and NK-92 cells, showed enhanced efficacy in targeting tumor spheroids by utilizing the interaction between NKG2D and its ligands MHC-I chain-related protein A and B^62^. Interestingly, based on the cytolytic effect of NK cell-derived exosomes, their use as a DDS in cancer immunological applications has been remarkably attractive. Han et al. demonstrated that NK cell-derived exosomes loaded with PTX showed a significantly enhanced antitumor effect on breast cancer compared to PTX treatment alone. This study suggests that these exosomes can serve as competitive tools for drug delivery^29^. Additionally, surface-engineered NK cell-derived exosomes can enhance their targeting capability and binding affinity toward other substances, consequently augmenting their duration of action and stability in vivo. In a study by Nie et al., NK cell-derived exosomes conjugated on the CTL surface were released at low pH in the TME, leveraging their tumor-targeting capability and promoting the cytotoxic action of CTLs against tumors^63^. In another study, a cocktail therapy strategy was employed, combining NK cell-derived exosomes with dendrimer cores loaded with therapeutic miRNAs, thus enabling targeted tumor therapy^64^. These innovative strategies underscore the potential of NK cell-derived exosomes in improving tumor-specific treatments. Altogether, these studies imply the high utility of NK cell-derived exosomes in advancing cancer immunotherapy, highlighting the efficacy of modified exosomes as valuable tools for cancer treatment.

### Macrophage-derived exosomes

Macrophages can undergo polarization into two distinct phenotypes upon exposure to environmental signals, namely, pro-inflammatory M1 and anti-inflammatory M2 macrophages^65,66^. M2 macrophages, which secrete immunosuppressive cytokines, including IL-4, IL-10, IL-13, and TGF-β, contribute to the inhibition of T cell immunity in the TME and increase tumor growth^67,68^. Conversely, M1 macrophages show tumoricidal activity through the secretion of tumor-killing molecules, such as reactive oxygen species (ROS), inducible nitric oxide synthase, and immunostimulatory cytokines, including TNF-α, IFN-γ, and IL-12^69–71^. Considering their noteworthy features, exosomes derived from M1 macrophages have gained prominence as influential entities in cancer immunotherapy. It has been reported that M1 macrophage-derived exosomes, which have absorbed antigens, can subsequently deliver them to CD4^+^ or CD8^+^ T cells through receptor‒ligand interactions promoted by specific surface ligands and adhesion molecules such as tetraspanins, phosphatidylserine, and ICAM-1^72^. Besides, these exosomes can serve as adjuvants for antitumor therapeutic agents, augmenting their efficacy in cancer treatment. M1 macrophage-derived exosomes demonstrated a distinctive ability to target lymph nodes, where they were selectively internalized by local macrophages and DCs. This led to the activation of Th1 inflammatory immune responses within the localized microenvironment^73^. Considering the transient and plastic nature of macrophages, Choo et al. devised M1 exosome mimetics to successfully reprogram M2 macrophages into the M1 phenotype, leading to a notable improvement in the therapeutic efficacy of anti-PD-L1 therapy^73^.

To increase the therapeutic efficacy of these exosomes in cancer treatment, a multitude of approaches are currently being investigated to modify exosomes derived from M1 macrophages. In the context of cancer vaccines, exosomes derived from M1 macrophages stimulated with IFN-γ play a crucial role in increasing antitumor activity. These exosomes contributed to building a pro-inflammatory microenvironment within the lymph nodes, thereby enhancing the efficacy of cancer vaccines^23^. Furthermore, a study highlighted that exosomes derived from M1-polarized macrophages exhibited the capacity to encapsulate PTX. These PTX-loaded M1 exosomes have shown the ability to enhance antitumor efficacy by establishing a proinflammatory microenvironment. This enhanced inflammatory milieu led to the upregulation of the expression of caspase-3, which is a pivotal mediator of apoptotic cell death, in breast cancer cells^30^. To impart a targeting ability to PTX-loaded M1 macrophage-derived exosomes, Kim et al. conjugated PTX-loaded M1 macrophage exosomes with an AA-PEG vector moiety that specifically recognizes sigma receptors overexpressed on specific lung cancer cells. The modified exosomes demonstrated enhanced efficacy in cancer treatment and significantly improved survival outcomes in mice with lung metastases^37^. Gunasekaran et al. modified M1 macrophage-derived exosomes with NF-κB p50 siRNA and miR-511-3p to induce M1 polarization and decorated the exosome surface with an IL4R binding peptide (IL4RPep-1) to specifically target IL-4 receptors on M2 macrophages. These modified exosomes, referred to as IL4R-Exo (si/mi), were absorbed by M2 macrophages, which downregulated the target genes, reduced M2 marker expression and increased M1 marker expression^74^. In an interesting study conducted by Wang et al., a novel approach was employed for chimeric exosome generation. These exosomes were derived from macrophage-tumor hybrid cells (aMT-exos), where M1 macrophages phagocytosed tumor cell nuclei. The unique characteristic of aMT-exos involves their tropism toward both lymph nodes and tumors. These modified exosomes displayed the ability to facilitate T cell activation and proliferation through direct exosome stimulation and traditional APC-mediated stimulation. The cumulative effect of these dual pathways led to the augmentation of anti-PD-1 antibody treatment efficacy, illustrating the potential of aMT-exos as a valuable tool in enhancing immunotherapeutic interventions^75^. Overall, these findings strongly indicate that modified M1 macrophage-derived exosomes hold great promise for immunoregulation and cancer therapy.

### Neutrophil-derived exosomes

Neutrophils, which are the predominant leukocytes in circulation, perform a variety of functions through the release of cytotoxic enzymes and effector molecules, ROS synthesis, phagocytosis and the generation of neutrophil extracellular traps^76^. Although the body of knowledge regarding exosomes released from other immune cells is continuously increasing, there remains a significant gap in understanding the specific contributions of neutrophil-derived exosomes. The limited exploration of these exosomes can be attributed to challenges associated with the short lifespan of neutrophils and the complexities involved in their isolation and manipulation^77^. Nonetheless, with the growing body of research on the pro-inflammatory role of IEXs, recent findings have shed light on the potential of neutrophil-derived exosomes as promising therapeutic agents in cancer therapy^78,79^.

On the basis of the spatiotemporal production mechanism, neutrophil-derived exosomes can be categorized into neutrophil-derived microvesicles (NDMVs) and neutrophil-derived trails (NDTRs)^80^. NDMVs are generated by neutrophils present at the site of inflammation, whereas NDTRs originate from neutrophils migrating from blood vessels to inflamed tissue^81,82^. While they share a common ability to effectively kill bacteria through ROS and granules, they often present significant distinctions^83^. NDMVs, characterized by the presence of protease-enriched granules, mostly exert anti-inflammatory effects by suppressing the expression of inflammatory genes in various cell types, including monocyte-derived DCs, macrophages and NK cells^84–86^. NDMVs have been observed to promote M0 macrophage polarization toward an anti-inflammatory phenotype, whereas NDTRs induce their polarization toward a pro-inflammatory phenotype^80^. Recently, emerging reports have highlighted the utility of neutrophil-derived exosome modification to propose a novel paradigm in cancer immunotherapy. In particular, neutrophil-derived exosomes loaded with drugs have emerged as a promising therapeutic approach for brain tumors by exploiting their distinctive abilities to be recruited to inflammatory sites and effectively penetrate the blood‒brain barrier (BBB)^78,87^. Wang et al. demonstrated that neutrophil-derived exosomes carrying doxorubicin (DOX) can rapidly cross the BBB and migrate into the brain. Moreover, intravenous injection of these exosomes in a glioma-bearing mouse model efficiently inhibited tumor growth and increased the survival rate^78^. Additionally, Zhang et al. successfully developed a targeted approach to cancer treatment by loading neutrophil-derived exosomes with DOX and incorporating superparamagnetic iron oxide nanoparticles (SPIONs). This innovative strategy ensured targeted drug delivery, resulting in the inhibition of tumor growth in mouse models bearing liver, colon, or gastric cancer^79^. These strategies underscore the potential of neutrophils as newly recognized promising candidates in the market of IEXs for cancer treatment.

## Modification of adaptive IEXs

**Fig. 3:**
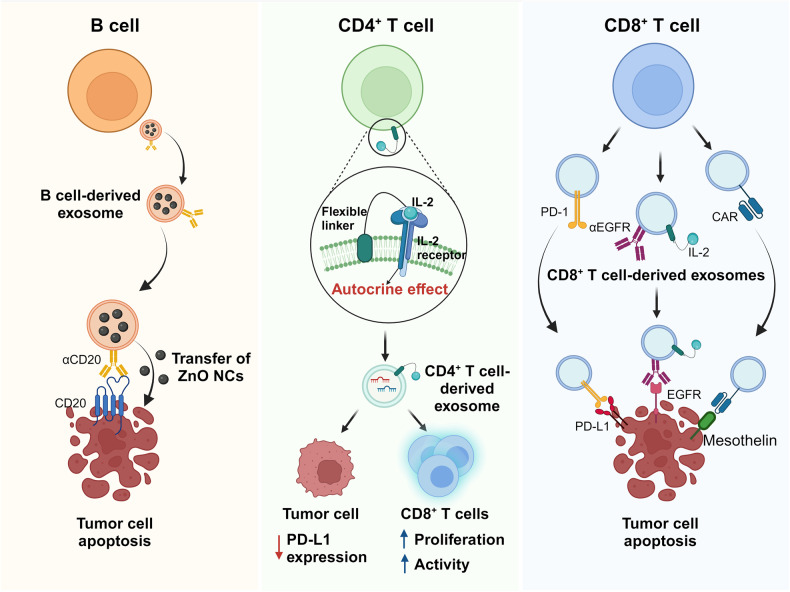
Antitumor effects of modified adaptive IEXs on tumor cells. Engineered exosomes derived from adaptive immune cells serve to enhance antitumor function. Left panel: Engineered B cell-derived exosomes, which display tumor-targeting anti-CD20 antibodies on their surface and load ZnO NCs, induce tumor-specific cell death. Middle panel: CD4^+^ T cell-derived exosomes tethered with IL-2 via MTFP amplify the antitumor efficacy through an autocrine effect. These exosomes diminish PD-L1 expression on tumor cells while increasing the proliferation and activity of CD8^+^ T cells. Right panel: Exosomes derived from CD8^+^ T cells expressing tumor-targeting PD-1, anti-EGFR antibodies, or CARs bind to tumor and induce tumor cell death. Created with BioRender.com.

Adaptive immunity consists of B cell-mediated humoral immunity and T cell-mediated cellular immunity, conferring the ability for the human body to mount targeted defenses against harmful agents, encompassing bacteria, viruses, toxins, and even cancer^88,89^. Similar to innate immune cells, adaptive immune cells also contribute significantly to antitumor immune responses^89,90^. Recently, there has been increasing interest in exploring the functions of adaptive IEXs within the cancer-immune system^18^. These exosomes have emerged as promising candidates for inducing antitumor immune responses, prompting extensive research into engineering strategies to augment their antitumor effects. In the following section, we aim to comprehensively review the antitumor functions of adaptive IEXs, including those derived from B cells and T cells, and examine various strategies involving modified exosomes for cancer therapy (Fig. 3 and Table 2).

**Table 2:** Application of modified adaptive IEXs for enhancing cancer treatment.

Source	Type of modification	Molecule involved in modification	Target cancer	Function	Reference
B cells	Heat shock	-	CT-26	- Induction of DC maturation, Th1 activity, and antigen-specific CTL response	^92^
	Freeze-thaw and incubation	ZnO NCs (freeze-thaw) and anti-CD20 antibody (incubation)	Daudi	- Increase in tumor-targeting ability - Reduction in viability of cancer cells	^94^
CD4^+^ T cells	Incubation	IL-2	B16F10 and SK-MEL-28	- Increase in CD8^+^ T cell proliferation and activity	^25^
	MTFP	IL-2	B16F10 and SK-MEL-28	- Increase in CD8^+^ T cell activity and proliferation - Downregulation of PD-L1 expressed on both cancer cells and their exosomes	^33^
CD8^+^ T cells	MTFP	IL-2 and anti-EGFR antibody	A549	- Enhancement of tumor-targeting ability - Reduction in exosome secretion in cancer cells - Augmentation of cancer cell cytotoxicity	^34^
	Lentiviral infection	PD-1	B16F10	- Neutralization of PD-L1 - Reinvigoration of CD8^+^ T cell activation and proliferation capacity - Induction of apoptosis in cancer cells	^103^
	Incubation	IL-12	-	- Activation of naive bystander CD8^+^ T cells	^26^
	Lentiviral infection	Mesothelin-targeted CAR	BT-549 and MDA-MB-231	- Augmentation of tumor-targeting ability - Elimination of cancer cells without side effects	^105^
	Lentiviral infection	EGFR-targeted CAR and HER2-targeted CAR	MCF-7, MDA-MB-231, MDA-MB-435, HCC827, and SK-BR-3	- Increase in tumor-targeting ability - Induction of cancer cell death without side effects	^106^

*Th1* T helper 1, *ZnO NCs* zinc oxide nanocrystals, *MTFP* membrane-tethering technology for proteins, *PD-L1* programmed death-ligand 1, *CAR* chimeric antigen receptor, *HER2* human epidermal growth factor receptor 2.

### B cell-derived exosomes

Beyond their role in specific immunoglobulin generation, B cells possess the capacity to modulate immune cell activities via various mechanisms, including antigen presentation, cytokine secretion and supportive costimulatory signals^90,91^. Similar to innate immune cells, B cells release exosomes that mimic the functional attributes of B cells in the immune system. Exosomes released from B lymphomas after heat shock exposure display augmented expression of immunogenic molecules such as MHC I, MHC II, CD40, CD86, RANTES, and IL-1β, with increases in HSP60 and HSP90^92^. These exosomes showed an elevated immunogenicity and delivered MHC complexes to induce CD4^+^ and CD8^+^ T cell proliferation and cytokine secretion, ultimately enhancing CTL responses^93^. In an intriguing investigation by Dumontel et al., B cell-derived exosomes were subjected to engineering with zinc oxide nanocrystals (ZnO NCs), which responded to external stimuli such as acoustic shock waves. The resulting engineered exosomes displayed remarkable cytotoxicity against Burkitt’s lymphoma cells. Furthermore, an anti-CD20 monoclonal antibody was integrated onto these exosomes to enable precise lymphoma cell targeting, further enhancing their therapeutic potential^94^. While limited studies have specifically evaluated the feasibility of utilizing modified B cell-derived exosomes in cancer treatment, such studies provide valuable insights into the enhancement of therapeutic potential through the modification of B cell-derived exosomes.

### CD4^+^ T cell-derived exosomes

T cells, which are pivotal immune cells in the body, play important roles in orchestrating and augmenting the immune response against pathogens, self-antigens, allergens, and cancers^95^. T cells can be categorized into various subsets, mainly cytotoxic CD8^+^ T cells and CD4^+^ T helper cells^95^. Among these subsets, CD4^+^ T cells play a vital role in immune modulation by engaging in interactions with other immune cells^96,97^. They primarily contribute to the antitumor immune response of CD8^+^ T cells and promote antibody production by B cells either through direct cell-to-cell interactions or IL-2 release^96,97^. With a recent interest in the field, CD4^+^ T cell-derived exosomes are considered promising agents that can mediate antitumor effects in cancer immunotherapy. In a study by Lu et al., it was revealed that CD4^+^ T cell-derived exosomes exerted a stimulating effect on humoral immune responses by enhancing the proliferation, activation, and antibody production of B cells^98^. This effect is mediated by exosome uptake facilitated by CD40L expressed on their surface.

As the understanding of the inherent antitumor roles of CD4^+^ T cells is increased, there have been emerging reports of studies focused on enhancing antitumor effects through the modification of CD4^+^ T cell-derived exosomes. Shin et al. reported that CD4^+^ T cell-derived exosomes enhanced the antitumor response of CD8^+^ T cells through miR-25-3p, miR-155-5p, miR-215-5p, and miR-375 without affecting regulatory T cells, resulting in the suppression of melanoma growth. Moreover, they found that IL-2 reinforced the antitumor efficacy of CD4^+^ T cell-derived exosomes^25^. In a study by Jung et al., IL-2-tethered exosomes were generated from engineered Jurkat T cells expressing IL-2 at the plasma membrane using a flexible linker to induce an autocrine effect. The surface engineering of IL-2 led to significant alterations in the miRNA profiles within these exosomes, resulting in the activation of CD8^+^ T cells and downregulation of PD-L1 expression in melanoma through differentially expressed miRNAs^33^. We suggest that these modified CD4^+^ T cell-derived exosomes can serve as novel activators of CD8^+^ T cells, offering a new approach to amplify antitumor efficacy in cancer immunotherapy.

### CD8^+^ T cell-derived exosomes

CD8^+^ T cells are a distinct population of white blood cells that exhibit their cytotoxic effects through the release of various cytokines. These cells play a pivotal role in specifically eliminating tumor cells, serving as a crucial defense mechanism in antitumor immunity^99^. According to multiple studies, CD8^+^ T cell-derived exosomes in cancer treatment undoubtedly show antitumor effects^26,100–102^. For instance, CD8^+^ T cell-derived exosomes possessed various cytotoxic molecules and either activated bystander T cells or suppressed lesional mesenchymal cells, establishing their multifaceted functional role^26,101^. Additionally, fully activated CTL-derived exosomes can promote the activation of CTLs with low affinity for antigens, thereby facilitating a comprehensive immune response^102^.

Most importantly, the modification of CD8^+^ T cell-derived exosomes has emerged as a thriving field of research within exosome-based cancer immunotherapy. In a recent study by Cho et al., it was revealed that engineered exosomes derived from primary CD8^+^ T cells expressing IL-2 and anti-EGFR antibodies not only demonstrated potent antitumor effects on A549 human lung cancer cells but also exhibited an augmented capacity for cancer targetability^34^. In light of the immunosuppressive role of PD-L1 expressed on cells or exosomes, Li et al. elucidated that exosomes derived from PD-1-expressing cytotoxic T cells had the capability to neutralize PD-L1, thereby increasing the activity and proliferation of CD8^+^ effector T cells and directly killing tumor cells through FasL and granzyme B^103^. Furthermore, IL-12-activated CTL-derived exosomes showed a unique capability to directly activate naive bystander CD8^+^ T cells, stimulating them to produce IFN-γ and granzyme B in the absence of antigens^26^. Moreover, CAR-T cells are highly esteemed in clinical studies as an emerging and effective modality for tumor treatment. Despite the remarkable potential of CAR-T cells as a systemic cell-based therapy, they have been associated with certain adverse effects, such as CRS^104^. Considering these factors, exosomes derived from CAR-T cells have emerged as a promising alternative to cell-based therapeutics for antitumor treatment. Yang et al. conducted a study in which they explored the potential of exosomes derived from mesothelin-targeted CAR-T cells for cancer immunotherapy. Interestingly, these exosomes revealed a striking resemblance to their parental cells, retaining crucial surface membrane molecules such as CARs, CD3, CD8 and T cell receptors (TCRs)^105^. Moreover, their therapeutic capacity was found to be comparable to that of the parental CAR-T cells, showing effective antitumor activity against triple-negative breast cancer cells without significant side effects. Additionally, the CAR-T cell-derived exosomes have been demonstrated to reduce CRS, thereby establishing them as a comparatively safer alternative to CAR-T cells^106^.

## Clinical application of the modified IEXs and challenges

Although IEXs offer numerous advantages, including their natural antitumor functions and ability to efficiently deliver therapeutic cargo, only a limited number of clinical trials have used these exosomes in cancer treatment. Among various IEXs, DEXs from cancer patients were found to be feasible and safe for immunotherapy in clinical trials. In a phase 1 clinical trial, melanoma-associated antigen gene-loaded DEXs showed modest responses and were well tolerated by patients with NSCLC or melanoma^107,108^. Subsequently, exosomes derived from mature DCs preconditioned by IFN-γ were used in a phase 2 clinical trial to enhance the limited T cell responses induced by DEXs^22^. In that trial, despite no notable changes in the levels or phenotype of circulating CD4^+^ and CD8^+^ T cells in peripheral blood, the administration of IFN-γ-matured DEXs increased NK cell activity in NSCLC patients after chemotherapy cessation. Although the clinical trial did not achieve the primary endpoint, defined as 50% of patients with progression-free survival after 4 months of chemotherapy cessation, the modified DEXs revealed remarkable antitumor responses and excellent tolerability, demonstrating greater potential for clinical application.

Despite the extensive interest on DEXs, IEX-based therapies still experience unresolved limitations that have hindered their widespread clinical application in cancer treatment^109^. Several hurdles need to be overcome for the clinical application of IEXs. First, it is necessary to address the issue of large-scale production of exosomes^110^. Although it has been reported that therapeutic DEXs can be prepared on a large scale through rapid purification, further testing is still required in different cell types^111^. Additionally, the establishment of cell culture and standardized exosome purification methods that can allow large-scale production of exosomes is crucial^112^. Second, the challenges in exosome storage, such as aggregation and cargo degradation during the freeze–thaw process, further limit their clinical use^113^. Even using the most comprehensive method of −80 °C cryopreservation, exosomes can undergo morphological changes, and biological activity may decline with long-term storage^114^. Therefore, there is an urgent need to study the long-term storage technology of exosomes to protect their biological activity and render them suitable for clinical applications. Third, exosomes derived from various types of cells are highly different in size, function, and composition, and this heterogeneity can be a major hurdle. Although several exosome isolation and characterization techniques have been developed to understand and resolve the heterogeneity in the biophysical properties and composition of exosomes, additional studies are needed to surmount their heterogeneity^114,115^. Employing IEXs for cancer therapeutics is attractive and promising. Additionally, modified IEXs have the potential to be an innovative therapeutic strategy; thus, more systematic studies to address the above challenges contribute to bringing these modified IEXs one step closer to their expanded clinical application.

## Conclusions and prospects

In this review, we introduce exosome modification strategies and provide a comprehensive overview of the therapeutic application of these modified IEXs in cancer treatment. Currently, extensive investigations into the structure, biogenesis, secretion, and function of exosomes have not only elucidated their significant role as mediators of cell-to-cell communication but also bolstered their potential as promising therapeutic agents^11^. In particular, IEXs possess several advantages, including excellent biocompatibility, low immunogenicity, high stability, and inherent antitumor activity, gathering significant interest in cancer therapeutics^4,11,13,15,18^. Simultaneously, efforts to enhance the efficacy of IEXs are underway through various strategies, including drug or antigen loading, surface engineering with targeting molecules and boosting exosomes with cytokine stimulation^5,17,22,31^. However, these methods still pose multiple challenges and need further optimization. Electroporation and sonication can cause exosome aggregation and membrane damage^19^. Genetic modification is confined to molecules suitable for genetic encoding, thus encompassing only a restricted range of molecules^17^. While chemical engineering can conjugate a broader range of molecules to exosomes, the surface complexity of exosomes may lead to decreased reaction efficiency and a lack of precise control of site specificity^17^. Thus, it is important to select the appropriate modification approach based on the drug to minimize the limitations. Collectively, owing to the ongoing developments in exosome engineering strategies, modified IEXs with enhanced antitumor effects and tumor-specific targeting show significant promise in advancing next-generation exosome-based cancer immunotherapy.
